# Complete response to Nivolumab-based chemotherapy in a case of advanced gastric cancer with multiple immune-related adverse events

**DOI:** 10.1007/s12328-026-02336-x

**Published:** 2026-04-27

**Authors:** Kazuto Takahashi, Hidetaka Matsuda, Yosuke Murata, Yu Akazawa, Tomoko Tanaka, Takuto Nosaka, Tatsushi Naito, Masahiro Otani, Yoshiaki Imamura, Yasunari Nakamoto

**Affiliations:** 1 Department of Gastroenterology, Second Department of Internal Medicine, Faculty of Medical Sciences, University of Fukui, 23-3 Matsuokashimoaizuki, Eiheiji-Cho Yoshida-Gun, Fukui, 910-1193 Japan; 2 Division of Diagnostic Pathology/Surgical Pathology, University of Fukui Hospital, Fukui, Japan

**Keywords:** Gastric cancer, Immune checkpoint inhibitors, Multi-organ immune-related adverse events, Therapeutic efficacy

## Abstract

Immune checkpoint inhibitors, such as nivolumab, have become pivotal in the treatment of advanced gastric cancer, particularly when combined with chemotherapy. While immune-related adverse events (irAEs) are commonly viewed as complications, their occurrence has been increasingly associated with favorable treatment responses. We report the case of a 59-year-old man diagnosed with advanced gastric cancer with liver and multiple lymph node metastases. The patient received chemotherapy, including nivolumab, but developed multiple grade 3 irAEs during treatment, including hypophysitis with secondary adrenal insufficiency, type 1 diabetes mellitus, and late-onset colitis. Despite these toxicities, imaging and endoscopy showed complete disappearance of the primary tumor and metastatic lesions, and a complete response was achieved per the Response Evaluation Criteria in Solid Tumors. Nivolumab monotherapy was continued for maintenance until gastrointestinal symptoms recurred due to irAE-associated colitis, which was successfully treated with corticosteroids. No tumor relapse was observed during this period. This case highlights that multi-organ irAEs are predictive of a favorable treatment response and that appropriate treatment of irAEs may result in excellent treatment outcomes in advanced gastric cancer.

## Introduction

Gastric cancer remains a major cause of cancer-related death globally, particularly in East Asia. Despite chemotherapy advances, unresectable or metastatic gastric cancer has a poor prognosis, with a median overall survival (OS) rarely exceeding 12 months when using conventional cytotoxic agents [1–3].

Immune checkpoint inhibitors (ICIs), especially PD-1 inhibitors like nivolumab, have shown promise in various malignancies. However, the response to ICIs has been modest in unselected populations of patients with gastric cancer. The ATTRACTION-2 trial reported an overall response rate (ORR) of only 11.2% with nivolumab monotherapy in pretreated advanced cases [4].

The phase III CheckMate 649 trial established nivolumab plus platinum and fluoropyrimidine-based chemotherapy as a first-line standard for HER2-negative advanced gastric, gastroesophageal junction, or esophageal adenocarcinoma. It demonstrated significantly improved OS (14.4 vs. 11.1 months) and progression-free survival (PFS) in patients with a PD-L1 combined positive score (CPS) ≥ 5 [5]. Although the overall complete response (CR) rate remained low, with approximately 12%, additional analyses subsequently showed that certain biomarkers were associated with a higher rate of achieving a CR or durable response. In the treatment of advanced gastric cancer, specifically, patients with high CPS, microsatellite instability (MSI)-high status, and Epstein-Barr virus (EBV)-positive tumors have been shown to have an improved response to ICIs [6, 7].

A unique feature of ICIs is the potential for immune-related adverse events (irAEs), which can affect multiple organs. Interestingly, irAEs have been linked to better outcomes. In gastric cancer, patients with irAEs during ICI therapy had longer OS and higher ORR compared to those without irAEs [8–10]. However, multi-organ irAEs remain rare.

Herein, we report a rare case of advanced gastric cancer that achieved durable CR with nivolumab-containing therapy despite the development of multiple irAEs involving the endocrine and gastrointestinal systems. This case highlights irAEs as possible markers of efficacy and underscores the importance of biomarker-guided therapy and irAE monitoring.

## Case report

In January 2022, a 59-year-old man presented to our hospital with epigastric pain. In March 2022, upper gastrointestinal endoscopy revealed an advanced tumor in the antrum of the stomach, with mixed histological findings of adenocarcinoma (tub1/pap/por). He had no underlying conditions. Contrast-enhanced abdominal computed tomography (CT) and fluorodeoxyglucose positron emission tomography identified multiple liver metastases and extensive lymphadenopathy, including involvement of the supraclavicular, mediastinal, periaortic, and perigastric lymph nodes. Laboratory examination revealed an elevated serum lactate dehydrogenase level of 621 U/L. Other hematological and biochemical parameters, including tumor markers, were within normal limits (Table 1). He was diagnosed with Union for International Cancer Control clinical stage IVB (T3N3M1) gastric adenocarcinoma, with HER2-negative and a PD-L1 CPS of ≥ 1 and < 5. Subsequent testing also revealed that the tumor was MSI-low and EBV-negative. He was diagnosed with Helicobacter pylori infection at the age of 51 and underwent eradication treatment.

**Table 1:** Laboratory data

*Peripheral blood*						*Serology*		
WBC	6900	/μL	Total protein	7.3	g/dL	CEA	2.4	ng/mL
Neutrophils	61.8%		Albumin	3.8	g/dL	CA19-9	7.1	U/mL
Eosinophils	1.6%		Total bilirubin	0.6	mg/dL	*Coagulation*		
Basophils	0.4%		AST	21	U/L	PT	97.1%	
Lymphocytes	28.0%		ALT	13	U/L	APTT	34.1	sec
RBC	452 × 10^4^	/μL	LDH	621	U/L	Fibrinogen	485	mg/dL
Hemoglobin	13.9	g/dL	ALP	83	U/L			
Hematocrit	43.0%		γ-GTP	21	U/L			
Platlet	32.7 × 10^4^	/μL	Amylase	81	U/L			
*Chemistry*			Glucose	79	mg/dL			
BUN	13	mg/dL	HbA1c	6.1%				
Creatinine	0.92	mg/dL						

WBC, white blood call; RBC, red blood cell; PT, prothrombin time; APTT, activated partial thromboplastin time; BUN, blood urea nitrogen; AST, aspartate aminotransferase; ALT, alanine aminotransferase; LDH, lactate dehydrogenase; ALP, alkaline phosphatase; γ-GTP, γ-Glutamyl transpeptidase; HbA1c, hemoglobin A1c; CRP, c-reactive protein; CEA, Carcinoembryonic antigen; CA19-9, carbohydrate antigen 19–9

In April 2022, he commenced first-line systemic therapy with a combination of nivolumab, S-1 (tegafur/gimeracil/oteracil), and oxaliplatin (SOX regimen). Two months later, after four cycles of chemotherapy, radiological evaluation and endoscopy showed a partial response with shrinkage of the primary lesion and metastatic sites.

In June 2022, the patient reported new-onset diarrhea and fatigue and was hospitalized. the patient reported new-onset diarrhea and fatigue. Early morning fasting blood samples showed low cortisol levels of < 1.0 μg/dL and ACTH levels of < 1.5 pg/mL. Laboratory and endocrine evaluation revealed grade 3 hypophysitis with secondary adrenal insufficiency, which was managed effectively with hydrocortisone replacement therapy. The development of diarrhea was determined to be due to S-1. Therefore, in July 2022, the chemotherapy regimen was modified to nivolumab plus CapeOX (capecitabine and oxaliplatin). He subsequently received 14 cycles of the modified regimen without further complications.

In March 2023, the patient developed fatigue, polydipsia, and polyuria. Laboratory evaluation revealed severe hyperglycemia (random blood glucose: 501 mg/dL, hemoglobin A1c: 8.2%), and the patient was admitted to the hospital. Also, anti-GAD antibody was negative. He was diagnosed with type 1 diabetes mellitus, which was presumed to be immune-mediated (grade 3 irAE), and he was started on intensive insulin therapy.

In April 2023, follow-up endoscopy and CT imaging showed complete resolution of the primary gastric tumor and hepatic metastases, along with a marked reduction in lymph node involvement. According to the Response Evaluation Criteria in Solid Tumors criteria, the patient achieved a CR. Nivolumab monotherapy was continued as a maintenance treatment until March 2024.

**Fig. 1:**
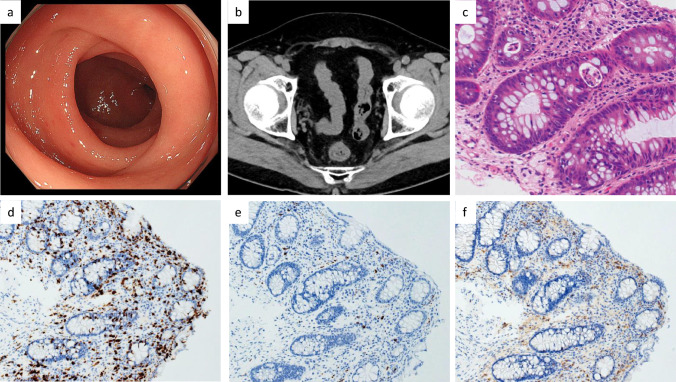
Imaging findings at the onset of irAE colitis. **a** Endoscopic imaging of the sigmoid colon revealed widespread edematous and erythematous mucosa. **b** A computed tomography scan also confirmed that edematous wall thickening had spread from the descending colon to the sigmoid colon. **c** Microscopic examination (200 × magnification) of H&E-stained sections of sigmoid colon biopsy specimens revealed inflammatory cell infiltration into the lamina propria and the formation of crypt abscesses. Immunostaining images (100 × magnification) revealed that the infiltrating cells were CD3 + T lymphocytes **d**, while CD4 **e** and CD8 **f** immunostaining revealed a predominance of CD8 + T lymphocytes

**Fig. 2:**
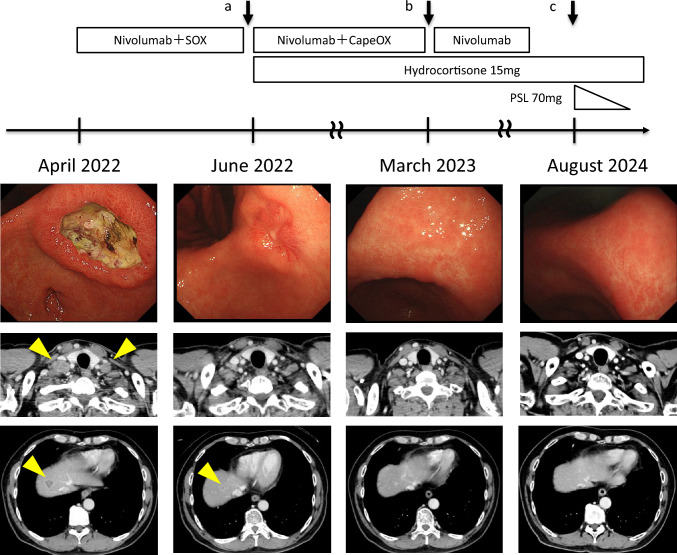
Clinical course with typical endoscopic and computed tomography images. The upper endoscope images show the primary gastric lesion, the middle images show the supraclavicular lymph nodes (yellow arrowhead), and the lower images show the liver metastasis (yellow arrowhead). The series of the three types of images all show the shrinking and disappearance of the lesions. The patient developed hypophysitis with secondary adrenal insufficiency as a grade 3 immune-related adverse event (irAE) at point a, type 1 diabetes (grade 3 irAE) at point b, and colitis (grade 3 irAE) at point c SOX, S-1 (tegafur/gimeracil/oteracil), and oxaliplatin; CapeOX, capecitabine and oxaliplatin; PSL, prednisolone

In August 2024, the patient presented with watery diarrhea occurring up to eight times per day. Stool cultures were negative for infectious organisms. Total colonoscopy revealed edematous and erythematous mucosa extending from the descending colon to the rectum. Histological analysis of biopsy specimens showed chronic inflammatory cell infiltration in the lamina propria and crypt abscess formation, predominantly composed of CD8-positive T lymphocytes, consistent with grade 3 immune-related colitis (Fig. 1). The patient was hospitalized and oral prednisolone was initiated at a dose of 1 mg/kg (70 mg/day). His symptoms improved rapidly, and the steroid dosage was gradually tapered and then discontinued. He was discharged in good condition, and no recurrence of malignancy was observed during follow-up to June 2025 (Fig. 2).

## Discussion

Nivolumab, an anti–PD-1 antibody, has emerged as a key component in the first-line treatment of unresectable or metastatic gastric and gastroesophageal junction adenocarcinomas. The pivotal phase III CheckMate 649 trial demonstrated that nivolumab combined with chemotherapy significantly prolonged OS and PFS compared with chemotherapy alone, particularly in patients with a PD-L1 CPS ≥ 5 [5]. Although our patient did not have high PD-L1 CPS, MSI-high status, or EBV-positivity, nivolumab plus chemotherapy was selected because it is an internationally accepted first-line standard regimen for HER2-negative advanced gastric cancer, as established by the CheckMate 649 trial. This regimen is recommended for patients with CPS ≥ 1, including our patient, and is considered an appropriate evidence-based option. In this case, the patient achieved a durable CR, despite presenting features not typically associated with a deep response to immunotherapy. In this case, the patient had a CPS between 1 and 5 and MSI-low disease, characteristics not typically associated with deep responses to immunotherapy. However, the patient achieved a durable CR.

One of the striking features of this case was the development of multiple irAEs, including hypophysitis, type 1 diabetes mellitus, and colitis, all grade 3 toxicities involving distinct organ systems. IrAEs result from overactivation of the immune system against normal tissues and can occur in any organ. The endocrine system is one of the most frequently affected, particularly the thyroid, pituitary, and pancreas. Hypophysitis and type 1 diabetes are less common but well-recognized irAEs, often leading to permanent hormonal insufficiency requiring lifelong replacement therapy [11, 12]. In this patient, endocrine irAEs were successfully managed with hormone replacement, allowing immunotherapy to be continued.

Importantly, as previously mentioned, several retrospective studies have reported a positive correlation between the occurrence of irAEs and improved clinical outcomes. Furthermore, Zhang et al. analyzed irAE profiles across patients with gastric cancer and concluded that the development of multi-organ irAEs was correlated with superior tumor control and prolonged survival in patients receiving PD-1/PD-L1 inhibitors. Specifically, a subgroup analysis revealed that patients who developed two or more grade ≥ 2 irAEs had significantly higher objective response rates and longer PFS than those with no or single-site irAEs [13]. In addition, the pattern of multi-organ irAEs significantly influences survival outcomes in patients receiving ICIs. Specifically, the development of endocrine and cutaneous irAEs is associated with improved OS, suggesting a favorable response to ICI therapy [14]. The exact mechanisms linking irAE development to tumor response are still under investigation but may reflect a more robust or sustained immune activation, capable of attacking both neoplastic and normal tissues.

Recent multicohort and retrospective studies have described clinical patterns and candidate risk factors for multi-organ irAEs, including older age, higher baseline albumin, smoking history, certain tumor types (e.g., thymoma), and early increases in peripheral eosinophils [14–17]. In our patient, despite the development of three severe irAEs, no clear pre-treatment clinical risk factor was apparent, suggesting that multi-organ toxicity can occur unpredictably and may reflect robust treatment-induced immune activation, which in this case coincided with durable tumor control.

Notably in this case, despite the presence of nonfavorable biomarkers (MSI-low, low CPS, EBV-negative), the patient’s immune system mounted a durable and deep response. He developed three distinct and severe irAEs—hypophysitis, type 1 diabetes mellitus, and colitis—yet achieved a durable CR lasting over two 2 years without disease relapse. This clinical course aligns with the hypothesis that the breadth of irAEs may parallel the intensity and effectiveness of antitumor immune responses, suggesting that multi-organ irAEs may be a powerful predictive marker, similar to tumor PD-L1 and MSI status.

Appropriate and timely management may allow for the continuation of immunotherapy despite the occurrence of irAEs. Continuation of nivolumab without discontinuing ICIs may have contributed to the sustained disease control in our patient. Understanding the balance between therapeutic benefit and immune toxicity remains critical for optimizing the use of ICIs. We did not restart nivolumab because the patient had already experienced multiple grade 3 irAEs, including colitis requiring steroid treatment, and the risk of severe recurrence was considered high. As the patient has maintained a durable complete response, observation without further immunotherapy was judged safest. If recurrence occurs, we would select the treatment based on the pattern of relapse. Chemotherapy would be considered first, while nivolumab rechallenge would be approached very cautiously due to the prior severe irAEs.

Lastly, conversion surgery has been proposed as a strategy following CR in patients with advanced gastric cancer [18]. Although conversion surgery is considered for selected patients who achieve a major response after systemic therapy, its benefit is mainly supported in those with limited metastatic burden. In our patient with initially widespread metastases, nivolumab-based therapy showed favorable effects from the start, resulting in a durable complete response maintained for more than two years. Therefore, we judged that continued immunotherapy and careful surveillance were more appropriate than surgery. However, long-term CR was maintained in this case without the use of surgery. This suggests that durable remission with ICI alone is possible if immune activation is profound and sustained, even in stage IV disease.

## Abbreviations

OS: Overall survival
ICIs: Immune checkpoint inhibitor
PD-1: Particularly programmed death-1
ORR: Overall response rate
PFS: Progression-free survival
CPS: Combined positive score
CR: Complete response
MSI: Microsatellite instability
EBV: Epstein–Barr virus
irAE: Immune-related adverse event
CT: Computed tomography
